# Clinical Cost-Saving Clues: A Case of Diagnosing a Rare Hypothyroid Myopathy

**DOI:** 10.7759/cureus.112705

**Published:** 2026-07-15

**Authors:** Andrea M Lauffer, Pascal Aliihnui Atanga, Sindhuri Benjaram, Poojitha Pathakamudi, Muhammad Anwar

**Affiliations:** 1 Hospital Medicine, Thomas Memorial Hospital - West Virginia University, South Charleston, USA; 2 Neurology, Thomas Memorial Hospital - West Virginia University, South Charleston, USA

**Keywords:** clinical medicine, cost-effective practice, creatine kinase, hoffmann syndrome, overt hypothyroidism

## Abstract

Severe hypothyroidism left untreated can lead to the progression of a distinct hypothyroid myopathy. This unique thyroid myopathy is characterized by weakness of the proximal limb muscles with an increase in muscle mass, muscle stiffness, and cramps. There is no consistent concept or guidelines for diagnostic work-up with the typical findings of this specific myopathy. While muscle biopsy can provide additional clinical information, the majority of cases can be diagnosed clinically by the hallmark signs of muscular weakness and pseudohypertrophy of muscles in combination with elevated creatine kinase (CK) and thyroid-stimulating hormone (TSH) levels. We present a patient who developed the hallmark findings of this rare hypothyroid myopathy, in which physical exam and laboratory data provided the diagnosis. Magnetic resonance imaging or muscle biopsy could have been performed to confirm the presence of muscular pseudohypertrophy, but the cost of testing would not have altered the treatment course. Therefore, the diagnosis can be cost-effectively made without invasive diagnostic and radiologic measures.

## Introduction

Untreated hypothyroidism is known for its classic associated symptoms that include constipation, weight gain, dry skin, and, in severe cases, myxedema coma. The clinician should also be aware of the associated debilitating myopathy that can present with severe hypothyroidism. Patients with Hoffmann's syndrome develop unique clinical findings, which include proximal muscle weakness and pseudohypertrophy of muscles [1]. Afflicted patients can experience gradual deconditioning and ambulatory dysfunction that may increase their risk of falls and need for aggressive physical rehabilitation therapy. Hoffmann first described the clinical features of this disease in 1897 after examining an adult patient who had muscular pseudohypertrophy following a thyroidectomy [2]. He described the myopathic and neurologic symptoms as stiffness and delayed relaxation of the deep tendon reflex, respectively [2]. There has been limited discussion in the literature about the approach for diagnosing Hoffmann's syndrome. Given the growing importance of practicing cost-effective medicine, this case reviews the approach to diagnosing this unusual hypothyroid myopathy in which invasive and expensive diagnostic modalities were not needed to reach a clinical conclusion. Even in the 21st century, the clinical clues discovered by Hoffmann can be used in the diagnosis of this rare myopathy. This case highlights the importance of recognizing myopathy as a potential significant symptom in cases of overt hypothyroidism, the physical exam clues associated with Hoffmann's syndrome, and the diagnostic approach that allows the implementation of radiologic and surgical stewardship, ultimately providing cost-saving practices for patients and medical institutions.

## Case presentation

**Figure 1:**
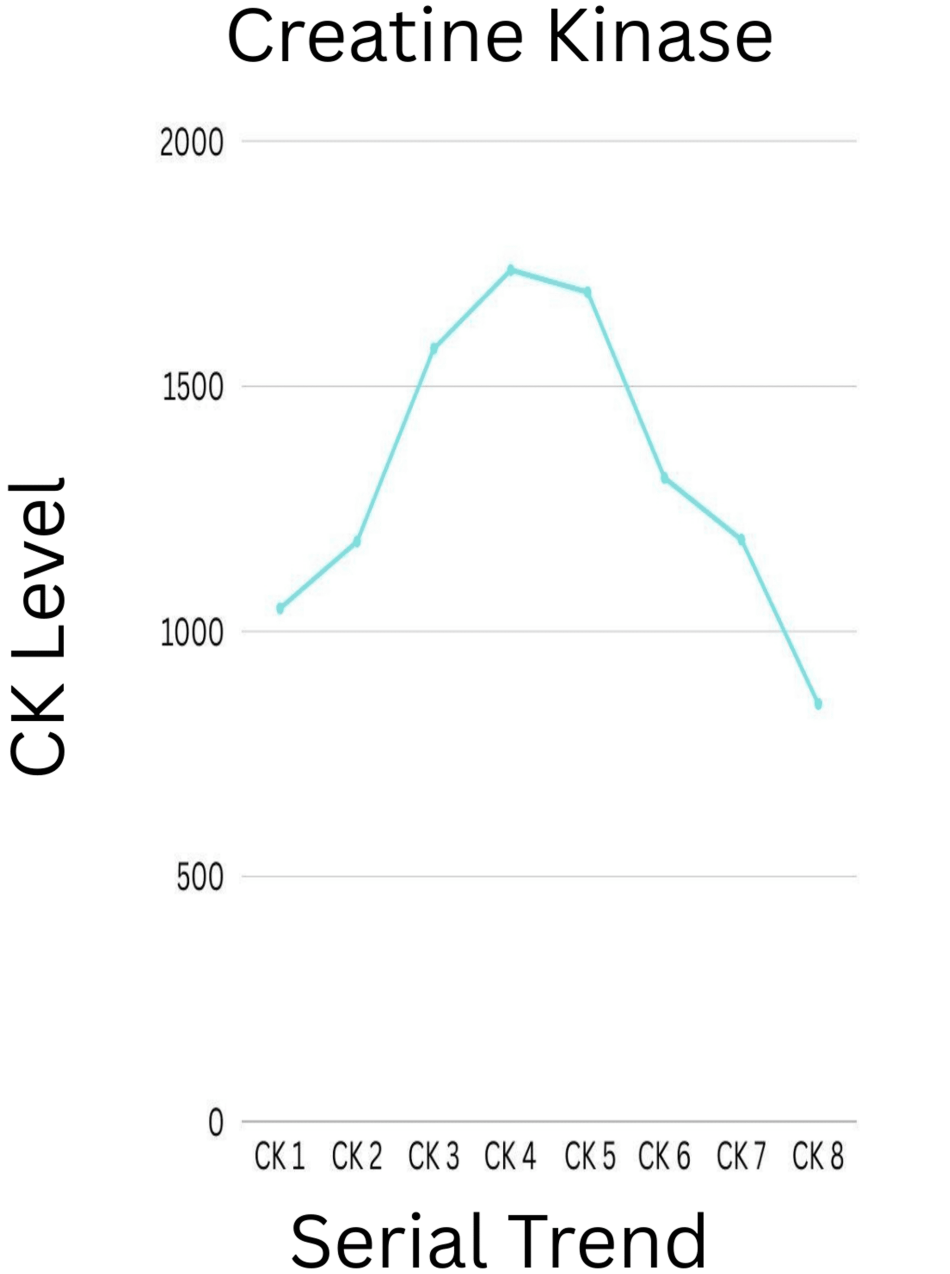
CK trend after hospitalization and the initiation of levothyroxine CK: creatine kinase

A 61-year-old male presented to the emergency department after he was unable to get up from his home recliner. He had a past medical history of hypothyroidism, obesity, chronic kidney disease stage 3, hypertension, and renal cell carcinoma status post nephrectomy and adrenalectomy. The patient had been experiencing myalgia and weakness in his lower extremities that had been ongoing for several months. In the emergency department, he complained of pain in his bilateral hips and ankles. Upon further questioning, the patient admitted to not seeing his physician in over a year. He was taking one home medication at the time of admission, which was propranolol. On physical exam, he had severe pain with active and passive movement of his lower extremities. His lower extremities were stiff. He had painful weakness of his bilateral hip flexors. There were +1 deep tendon reflexes noted overall. Muscle bulk was difficult to assess due to pain and the presence of concurrent obesity. Initial creatine kinase (CK) level was 1,047 U/L (Table 1). Thyroid-stimulating hormone (TSH) was 59.3 uIU/mL (Table 1). Free thyroxine hormone (T4) was < 0.10 ng/dL. He was started on intravenous levothyroxine 100 mcg daily and received aggressive IV fluid hydration. He was also given a stress dose of steroids due to his history of adrenalectomy. During his hospital admission, his CK level peaked to 1,737 U/L (see Figure 1). Neurology was consulted to evaluate for underlying myopathy. Given his physical exam and laboratory findings, the neurologist clinically concluded that the patient's myopathy was due to Hoffmann's syndrome, which occurred due to the patient's prolonged non-compliance in treating his underlying hypothyroid disease. During the patient's hospital course, his muscle pain gradually improved. Ten days after his initial presentation, his TSH was 50.8 uIU/mL, and free T4 was 0.84 ng/dL. His overall hospital course was protracted due to the slow recovery of his functional mobility (Table 2). He was transitioned from intravenous to oral levothyroxine. The patient was discharged to a skilled nursing facility for further rehabilitation. 

**Table 1:** Thyroid studies and CK results CK: creatine kinase; TSH: thyroid-stimulating hormone; T4: thyroxine hormone

Lab	Reference Range	Hospital Day 1	Hospital Day 2	Hospital Day 3	Hospital Day 4
TSH	0.270-4.2 uIU/mL	59.3			
Free T4	0.93-1.70 ng/dL	<0.10 ng/dL			
CK	< = 190 U/L	1,047 —> 1,183 —> 1,577	1,737 —> 1,692	1,313 —> 1,187	852

**Table 2:** Timeline of the patient's clinical course CK: creatine kinase; TSH: thyroid-stimulating hormone; T4: thyroxine hormone

Hospital Day	Event
1	Day of admission. Patient complains of myalgia, inability to ambulate, and leg stiffness. Elevated TSH and CK (59.3 uIU/mL and 1183 U/L) noted. Endocrine consulted and oral levothyroxine started.
2	Leg stiffness lessened. Neurology consulted to address myopathy. Endocrine transitions levothyroxine from oral to intravenous.
3	Leg stiffness lessened, but present. Neurology makes clinical diagnosis of Hoffmann’s syndrome. Endocrine makes note of improving CK level.
4	Worsening myalgia when working with physical therapy/occupational therapy (PT/OT). Final CK check of hospitalization (852 U/L).
5	Patient transitioned to PO levothyroxine.
6	Continues to work with PT/OT; skilled nursing referrals (SNF) made.
7-13	Participated in PT/OT and eventually discharged to SNF.

## Discussion

Hoffmann's syndrome is a rare form of hypothyroid myopathy first characterized by German neurologist Johann Hoffmann [1]. An analogous condition exists for children born with congenital hypothyroidism, which is known as Kocher-Debre-Semelaigne syndrome [2]. Almost one-third of patients who present with hypothyroidism have muscular complaints, but Hoffmann's syndrome is unique in its clinical presentation [2]. Accumulation of glycosaminoglycans leads to pseudohypertrophy of the muscles [3]. Chronic thyroid insufficiency leads to a change in muscle fibers from fast to slow type that causes weakness and hyporeflexia [3]. The pathophysiology involves the effects of thyroid hormone deficiency on muscle metabolism, leading to abnormal muscle growth and functional deterioration [4]. It is characterized by weakness of the proximal limb muscles with an increase in muscle mass, muscle stiffness, and cramps [4]. It occurs most often in male adults with longstanding untreated hypothyroidism [5]. In a recent meta-analysis study, there was no consistent concept for diagnostic work-up with typical findings. While muscle biopsy can provide additional clinical information, the majority of cases were diagnosed clinically by the hallmark signs of muscular weakness and pseudohypertrophy of the calf muscles in combination with elevated CK and elevated TSH levels [5]. Of the cases that did have a muscle biopsy performed, the results were not uniform and described very differently [5]. None of the reports found any signs of inflammation, and one of the muscle biopsies was normal [5]. One unifying clinical exam finding in the meta-analysis was pseudohypertrophy of the calf muscles [5]. However, involvement of the gastrocnemius, deltoid, and trapezius muscles has been described in the literature as well [6]. CK level can be very elevated in thyroid myopathy, but it is not often correlated with the degree of weakness [6]. A complication particularly threatening to our patient, given his history of nephrectomy, is rhabdomyolysis, which could have caused renal damage to his existing solitary kidney [7]. Another diagnostic tool that has been used is electromyography (EMG), in which repetitive positive waves were revealed in a patient with Hoffmann's syndrome [8]. Overall, muscle hypertrophy with stiffness occurs in only < 10% of patients with hypothyroidism, which highlights the rarity of this diagnosis [8]. With regard to our patient, he met the clinical diagnostic criteria for Hoffmann's syndrome. It was difficult to ascertain the presence of pseudohypertrophy of the muscles due to his body habitus. Magnetic resonance imaging or EMG could have been performed to confirm the presence of pseudohypertrophy and abnormal muscle waves, but the cost of testing would not have altered the treatment course.

## Conclusions

Hypothyroidism is a common disease encountered both in the inpatient and outpatient settings. Many patients with hypothyroidism will present with muscle complaints in the absence of Hoffmann's syndrome. Therefore, understanding its unusual clinical and myopathic presentation and its correlation with profound hypothyroidism is essential.

Hoffmann's syndrome is a rare myopathy found often in males with profound hypothyroidism. While invasive and expensive diagnostic measures can be used for solidifying the diagnosis, the hallmark clinical exam findings of muscle pseudohypertrophy, muscle weakness, and muscle stiffness in combination with laboratory findings of elevated CK and TSH levels are the most cost-efficient and resourceful way of reaching the diagnosis and initiating prompt treatment.
